# Risk Factors for Norovirus, Sapporo-like Virus, and Group A Rotavirus Gastroenteritis

**DOI:** 10.3201/eid0912.020076

**Published:** 2003-12

**Authors:** Matty AS de Wit, Marion PG Koopmans, Yvonne THP van Duynhoven

**Affiliations:** * National Institute of Public Health and the Environment, Bilthoven, the Netherlands

**Keywords:** risk factors, viral gastroenteritis, norovirus, Sapporo-like virus, rotavirus, hygiene, food handling, person-to-person contact

## Abstract

Viral pathogens are the most common causes of gastroenteritis in the community. To identify modes of transmission and opportunities for prevention, a case-control study was conducted and risk factors for gastroenteritis attributable to norovirus (NV), Sapporo-like virus (SLV), and rotavirus were studied. For NV gastroenteritis, having a household member with gastroenteritis, contact with a person with gastroenteritis outside the household, and poor food-handling hygiene were associated with illness (population attributable risk fractions [PAR] of 17%, 56%, and 47%, respectively). For SLV gastroenteritis, contact with a person with gastroenteritis outside the household was associated with a higher risk (PAR 60%). For rotavirus gastroenteritis, contact with a person with gastroenteritis outside the household and food-handling hygiene were associated with a higher risk (PAR 86% and 46%, respectively). Transmission of these viral pathogens occurs primarily from person to person. However, for NV gastroenteritis, foodborne transmission seems to play an important role.

Recent studies in the Netherlands and other countries have shown that viral infections, especially noroviruses (NV), are the most frequent cause of gastroenteritis in the community, both outbreak-related and endemic (*1*–*8*). The overall incidence of gastroenteritis in the Netherlands was estimated at 283 per 1,000 persons per year in a community-based study in 1999 (*3*). NV was detected in 11% of cases, Sapporo-like viruses (SLV) in 2%, and rotavirus group A in 4%. The incidence at the general-practice level from 1996 to 1999 was estimated at 14 per 1,000 person-years; 5% was attributed to NV, 2% to SLV, and 5% to rotavirus (*2*,*9*). For rotavirus, preventive measures currently focus on developing vaccines to reduce hospitalizations in cases where the illness is complicated by dehydration. For caliciviruses, in spite of their high incidence, little effort has gone into prevention, and little is known about preventable routes of infection. Although possible transmission routes, such as food products or water supplies (*10*,*11*), were identified in outbreaks, sources in endemic cases are difficult to detect. Therefore, we conducted a case-control study to identify risk factors that could provide leads for preventing endemic cases of viral gastroenteritis attributable to caliciviruses and rotavirus.

## Methods

A community-based prospective cohort study with a nested case-control study was undertaken in the Netherlands in 1999 (*3*). The cohort was followed to estimate the incidence of gastroenteritis. The nested case-control study was used to identify risk factors and determine etiology. The study was performed in cooperation with the sentinel general practice network of the Netherlands Institute of Primary Health Care. The cohort consisted of an age-stratified sample of persons registered at general practices in this network. Cases identified in the community-cohort with gastroenteritis were included in the case-control study, and a matched control was selected from the cohort members without gastroenteritis at that time. Case-patients and controls were matched by age, degree of urbanization, region, and date of inclusion. At the start of follow-up, all persons in the cohort completed a questionnaire on demographic characteristics and long-term risk factors (such as food-handling practices and presence of animals). Case-patients and controls included in the case-control study completed a questionnaire addressing short-term risk factors in the 7-day period before onset of symptoms and submitted stool samples. Case-patients submitted four stool samples (on days 1, 8, 14, and 21 of the episode), and controls submitted two stool samples (on days 1 and 8 from inclusion as a control). Samples were tested for NV and SLV by reverse transcription-polymerase chain reaction and for rotavirus group A by enzyme-linked immunosorbent assay, as described (*3*,*12*–*14*).

### Potential Risk Factors

Potential risk factors we studied were chronic gastrointestinal symptoms, being breastfed, having animals in the household (both pets and farm animals), food-handling hygiene index, method of keeping and heating up leftover food, presence of household equipment (blender, dishwasher, microwave, freezer), child in diapers in household, participant or other child in household attending a daycare center or primary school, size of household, being pregnant, being vegetarian, nationality, country of birth of participant and parents, being employed, type of house, income, educational level, age, and sex. The following factors were studied in the week before onset of illness or before inclusion as a control: contact with others with gastroenteritis (in and outside the household); swimming or other water-related sports; foreign travel; use of antimicrobial drugs, consumption of (raw or well-done) chicken, pork, beef, organ meat, meat in dough, fish, crab, shrimp, oysters, mussels, raw vegetables, salad, fruits, dried fruits, rice, raw milk, ice cream, soft cheeses, runny eggs, raw eggs, take-away fast-food, take-away bread rolls, take-away kebab, take-away Chinese food, meal services, food from canteen, food from reception, food from barbecue, eating out in a restaurant, and contact with farm animals (with or without diarrhea).

### Statistical Analyses

All gastroenteritis case-patients who tested positive in the first or second stool samples and their matched controls were included in the analyses. Gastroenteritis was defined as one of the following: three or more loose stools in 24 hours; three or more vomiting episodes in 24 hours; diarrhea with at least two additional symptoms; or vomiting with at least two additional symptoms. Additional symptoms were abdominal pain, abdominal cramps, nausea, blood in stool, mucus in stool, fever, diarrhea, or vomiting.

Univariate analyses were completed by using McNemar and Bowkers test for symmetry for categorical variables and paired t tests and Wilcoxon signed rank test for continuous variables. A conditional logistic regression model was used to study the independent effects of risk factors with an association in the univariate analyses with a p value of <0.10. Selection of variables in the model was backwards manually, based on the log-likelihood ratio; a significance level of 0.05 was used.

All risk factors in the questionnaire were studied to have the possibility to generate hypotheses on transmission, in addition to confirming and clarifying existing theories. Since the specific variables on food handling in the questionnaires were mainly focused on possible risk factors for bacterial gastroenteritis, they were used as indicators of food-handling hygiene in these analyses. An index was made for food-handling hygiene on the basis of several indicator variables. Two different scores were developed: a basic score, calculated by adding up all factors and weighing them equally, and an optimized score, which used the β from a logistic model as the weight for each factor. This logistic model was fit on NV gastroenteritis as an outcome because this was the largest group. The following variables were included as indicators (factors marked with an asterisk were independent indicators in the optimized score): frequency of shopping, *checking the appearance of product in shop, checking the packaging for damage in shop, following the storage instructions, checking the expiration date, *duration of keeping eggs, *use of same cutting board for raw meat and other products, *washing of cutting board between use for raw meat and other products, and frequency of changing dish brush, *scourer, and dishcloth.

The effect of food-handling hygiene includes both the effect of poor food-handling hygiene in the household favoring indirect person-to-person transmission and foodborne infection by introduction of contaminated food into the household. To estimate the second effect separately, we estimated the proportion preventable by hygienic food handling among those not in contact with other persons with gastroenteritis in the last week. This estimate was made by calculating the incidence attributable to food-handling hygiene among those not exposed to other persons with gastroenteritis and dividing it by the total incidence of virus-specific gastroenteritis. We assumed that all persons who reportedly had contact with a person with gastroenteritis were infected by that person.

Because age was likely to interact with all variables, we constructed a separate model for the age groups <5 years and >5 years. This stratification was possible for NV only because not enough adults were infected with rotavirus and SLV to make the analysis.

Population-attributable risk fractions (PAR) were calculated on the basis of multivariate odds ratios (OR) by estimating the incidence attributable to the risk factor and dividing it by the total incidence of virus-specific gastroenteritis. The total incidence of virus-specific gastroenteritis was calculated by multiplying the proportion positive and the overall incidence of gastroenteritis in the cohort. The incidence attributable to the risk factor was calculated as the total virus-specific incidence minus the estimated incidence if the risk factor was absent, which was estimated by weighing the cases according to their exposure status. Exposed cases were weighed as 1/OR of exposure, nonexposed cases as 1. All incidence estimates were standardized by age and cohort. Data from the case-control study were extrapolated to the entire cohort.

## Results

### Norovirus (NV)

In total, 152 case-patients were positive for NV—57 in both stool samples, 57 only in the first sample, and 38 only in the second sample. Of the matched controls, seven were positive for NV but did not have gastroenteritis. The median age of case-patients was 2 years (age distribution: <1 year: 47 [31%]; 1–4 years: 60 [39%]; 5–9 years: 25 [16%]; 10–59 years: 12 [8%]; >60 years: 8 [5%]).

**Figure 1:**
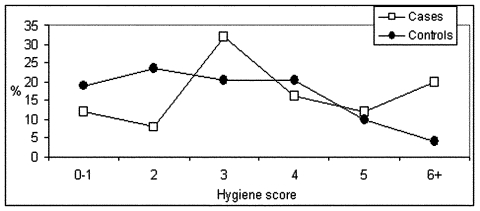
Distribution of basic food-handling hygiene score in norovirus gastroenteritis cases (n = 152) and controls (n = 152). (A higher score indicates less hygienic practices.)

NV gastroenteritis was independently associated with food-handling hygiene, having more than one household member with gastroenteritis (hereafter referred to as household gastroenteritis contact), and having contact with a person with gastroenteritis outside the household (hereafter referred to as outside gastroenteritis contact) in the week before onset of symptoms (Table 1, Figure 1). For the risk factor of household gastroenteritis contact, risk was slightly higher if the household member was a child rather than an adult (5.2 [95% confidence intervals (CI) 1.8 to 15.3] vs. 4.4 [95% CI 2.0 to 9.6]). Risks were comparable if the household member had diarrhea or vomiting. Because of the strong correlation of all variables on household contacts, only the number of household gastroenteritis contacts was included in the model. The contact of cases with symptomatic persons outside the household had taken place in the house of friends or family (31%), a daycare center (19%), school (18%), home (10%), or other places and work (22%). Cases and controls did not differ significantly. The association of attendance at daycare and primary school with NV gastroenteritis in the univariate analysis was no longer observed after correction for household gastroenteritis contact, especially if the sick household member was a child.

**Table 1:** Risk factors for NV gastroenteritis, prevalence in cases and controls (152 pairs), and univariate and multivariate odds ratios using logistic regression and population-attributable risk fractions^a^

NV gastroenteritis	Cases n (%)	Controls n (%)	OR uni	95% CI	OR multi	95% CI	PAR (%)	
Food-handling hygiene^b^			1.3	1.0 to 1.5	1.3	1.0 to 1.7	47	
Educational level					n.i.			
Low	21 (14.3)	16 (10.9)	1.9	0.9 to 4.0				
Intermediate	58 (39.5)	80 (54.4)	1.0	-				
High	68 (46.3)	51 (34.7)	2.2	1.2 to 3.9				
Participant to daycare center	47 (30.9)	37 (24.7)	1.7	0.9 to 3.3	n.i.			
Household member to daycare center	34 (23.5)	21 (14.5)	2.0	1.0 to 3.9	n.i.			
Household member to primary school	62 (42.8)	48 (33.1)	1.6	1.0 to 2.7	n.i.			
Pets in household	85 (56.3)	102 (67.6)	0.6	0.4 to 1.0	n.i.			
Cat as pet	46 (30.5)	61 (40.4)	0.6	0.4 to 1.0	n.i.			
No. of household members with gastroenteritis^c^							17	
None	73 (48.3)	130 (85.8)	1.0	-	1.0	-		
1	39 (25.8)	15 (10.0)	3.7	1.7 to 8.0	1.2	0.3 to 4.2		
>1	39 (25.8)	6 (4.2)	13.1	3.9 to 34.7	10.9	2.0 to 60.5		
Contact with persons outside household with gastroenteritis^c^							56	
No	50 (32.9)	101 (66.5)	1.0	-	1.0	-		
Yes	57 (37.5)	8 (5.3)	11.4	4.7 to 27.3	12.7	3.1 to 51.8		
Do not know	45 (29.6)	43 (28.3)	1.9	1.1 to 3.4	2.5	1.0 to 6.5		
Consumption of fish^c^	46 (34.6)	32 (24.1)	1.8	1.0 to 3.2	n.i.			
Consumption of barbecued food^c^	1 (1.5)	9 (6.6)	0.2	0.05 to 1.0	n.i.			

^a^NV, norovirus; OR, odds ratio; PAR, population-attributable risk fractions; uni, univariate; multi, multivariate; CI, confidence interval; n.i, not in final model; -, not applicable. ^b^Basic score (not optimized), higher score indicates less hygienic practices, OR for increase of 1. ^c^In the week before onset of symptoms (case-patients), inclusion in study (control).

Food-handling hygiene and outside gastroenteritis contact were the factors with the highest impact, as measured by PAR (Tables 1 and 2). PAR for all significant risk factors combined was 80%. PAR for outside gastroenteritis contact and household gastroenteritis contact combined accounted for 63% of cases. PAR for the two factors representing transmission in the household combined (hygiene and household gastroenteritis contact) was 56%. This figure is much lower than the sum of both PARs.

**Table 2:** Risk factors for NV gastroenteritis in persons <1 year to 4 years of age (n = 105 pairs) and persons >5 years of age (n = 46 pairs), univariate and multivariate odds ratios^a,b^

NV gastroenteritis	<1 y to 4 y (n = 105 pairs)					>5 y (n = 46 pairs)					
	OR uni	95% CI	OR multi	95% CI	PAR (%)	OR uni	95% CI.	OR multi	95% CI		PAR (%)
Food-handling hygiene	1.2	0.9 to 1.5	1.2	0.9 to 1.7	46	1.3	0.9 to 1.9	1.3	0.8 to 2.2		63
Household members with gastroenteritis					27						4
Yes	4.4	2.2 to 9.2	2.7	0.8 to 8.9		15.0	2.0 to 113.6	1.1	0.1 to 15.9		
No	1.0	-	1.0	-		1.0	-	1.0	-		
Contact with persons outside household with gastroenteritis					51						60
No	1.0	-	1.0	-		1.0	-	1.0	-		
Yes	17.7	5.1 to 61.1	10.9	2.2 to 54.6		5.9	1.0 to 20.1	12.1	1.0 to 147.3		
Do not know	2.4	1.2 to 4.7	2.7	0.9 to 7.8		0.8	0.2 to 3.0	1.8	0.2 to 15.3		

^a^Using logistic regression, and population-attributable risk fractions (PAR). ^b^NV, norovirus; OR, odds ratio; PAR, population-attributable risk fractions; uni, univariate; multi, multivariate; CI, confidence intervals; -, not applicable.

For persons >5 years of age, the effect of household gastroenteritis contact was reduced (OR = 1.1, PAR = 4%) when controlling for food-handling hygiene. Both factors (food-handling hygiene and household gastroenteritis contact) combined showed a similar PAR in both age groups (60% vs. 65%). Use of an optimized food-handling hygiene score for NV gastroenteritis resulted in a higher estimate of PAR for food-handling hygiene (60%).

We estimated the effect of contaminated food’s entering the household, separate from transmission from symptomatic contact persons through food to the patient, as explained in Methods. (We assumed that all case-patients who had contact with a person with gastroenteritis in the week before onset of symptoms were infected by these contacts.) Thirty-four pairs remained for the calculation of OR. For food-handling hygiene, OR was slightly higher for those not having contact with someone with gastroenteritis (1.4, 95% CI 0.8 to 2.2). The estimate of PAR for contaminated food’s entering the household was 12% of all NV gastroenteritis cases and 16% when the optimized score was used.

### Sapporo-like Viruses

In total, 48 cases were positive for SLV—21 in both samples, 22 only in the first sample, and 5 only in the second sample. Of the matched controls, two were positive for SLV but did not have gastroenteritis. The median age of SLV gastroenteritis case-patients was 1 year (age-distribution: <1 year: 19 [40%]; 1–4 years: 21 [44%]; 5–9 years: 4 [8%]; 10–68 years: 4 [8%]).

Outside gastroenteritis contact was the only independent risk factor for SLV gastroenteritis (Table 3). The location of contacts of case-patients and controls did not differ significantly. For case-patients, 29% of contacts took place at daycare, 21% at homes of friends or family, 14% at home, and 7% at school.

**Table 3:** Risk factors for SLV gastroenteritis, prevalence in cases and controls (48 pairs), univariate and multivariate odds ratios using logistic regression and PAR^a^

SLV gastroenteritis	Cases	Controls	OR uni	95% CI	OR multi	95% CI	PAR (%)	
	N (%)	N (%)						
Household member with gastroenteritis^b^	19 (39.6)	10 (21.3)	2.8	1.0 to 7.8	n.i.			
Contact with person outside household with gastroenteritis^b^							60	
No	1 (25.0)	28 (58.3)	1.0	-	1.0	-		
Yes	14 (29.2)	8 (16.7)	4.4	1.3 to 14.9	4.4	1.3 to 14.9		
Do not know	22 (45.8)	12 (25.0)	4.1	1.4 to 11.6	4.1	1.4 to 11.6		

^a^SLV, Sapporo-like virus; PAR, population-attributable risk fraction; OR, odds ratio; uni, univariate; multi, multivariate; CI, confidence interval; n.i., not in final model. ^b^In week before onset of symptoms.

### Rotavirus

In total, 54 cases were positive for rotavirus—11 in both samples, 41 only in the first, and 2 only in the second. None of the matched controls was positive for rotavirus. The median age of rotavirus gastroenteritis case-patients was <1 year (age distribution of cases: <1 year: 28 [52%]; 1–4 years: 18 [33%]; 5–9 years: 3 [6%]; 10–72 years: 5 [9%]).

**Figure 2:**
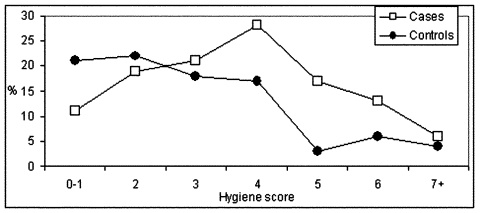
Distribution of food-handling hygiene score in rotavirus gastroenteritis cases (n = 54) and controls (n = 54). (A higher score indicates less hygienic practices.)

Rotavirus gastroenteritis was independently associated with outside gastroenteritis contact and with food-handling hygiene (Table 4 and Figure 2). A strong independent negative association was found with presence of a blender in the household. By univariate analysis, a high education level was a risk factor for rotavirus gastroenteritis, as was a household gastroenteritis contact. The risk was higher if the household gastroenteritis contact was a child (OR 5.8, 95% CI 1.3 to 25.6) than if the contact was an adult (OR 4.0, 95% CI 0.5 to 38.2). The association of a household gastroenteritis contact disappeared after correction for outside gastroenteritis contact. The association with educational level disappeared after correction for food-handling hygiene. Locations of outside gastroenteritis contacts did not differ significantly between cases and controls. For cases, 40% of contacts took place at homes of friends or family, 30% at daycare centers, and 20% at home. PAR for outside gastroenteritis contact was 86%; for food-handling hygiene, PAR was 46%. PAR for both factors combined was 92%. Use of the optimized food-handling hygiene score resulted in a PAR for food-handling hygiene of 64% and PAR for all factors combined of 96%.

**Table 4:** Risk factors for rotavirus gastroenteritis, prevalence in cases and controls (54 pairs), univariate and multivariate odds ratios using logistic regression and PAR^a^

Rotavirus gastroenteritis	Cases		Controls		OR uni	95% CI	OR multi	95% CI	PAR (%)	
	N (%)		N (%)							
Household member with gastroenteritis							n.i.			
No	30 (63.4)		44 (91.7)		np					
Yes, 1	10 (21.3)		4 (8.3)							
Yes, >1	7 (14.9)		0 (0.0)							
Contact with persons with gastroenteritis outside household									86	
No	13 (24.1)		33 (61.1)		1.0	-	1.0	-		
Yes	10 (18.5)		6 (11.1)		6.4	1.5 to 27.5	12.9	1.2 to 133.6		
Do not know	31 (57.4)		15 (27.8)		8.2	2.3 to 29.0	14.8	1.8 to 120.6		
Educational level							n.i.			
Low	2 (3.7)		6 (11.1)		0.3	0.0 to 2.9				
Middle	15 (27.8)		23 (42.6)		1.0	-				
High	37 (68.5)		25 (46.3)		2.1	0.9 to 4.6				
Food-handling hygiene score^b^					1.2	1.0 to 1.6	1.5	1.1 to 2.1	46	
Blender in household	16 (29.6)		30 (55.6)		0.2	0.1 to 0.7	0.1	0.0 to 0.6		

^a^OR, odds ratio; PAR, population-attributable risk fraction; uni, univariate; multi, multivariate; CI=confidence interval; np, not possible to calculate; n.i.: not in final model. ^b^Higher score indicates less hygienic practices, OR for increase of 1.

We estimated the effect of contaminated food’s entering the household in the same way as we did for NV gastroenteritis. The case-patients in 10 pairs had not had any contact with a person with gastroenteritis. In this group, OR for food-handling hygiene was 1.8 (95% CI 0.8 to 3.9), which is higher than in the total group of rotavirus cases. The estimate of PAR for contaminated food’s entering the household, based on these assumptions, was 4% of all rotavirus gastroenteritis cases.

## Discussion

To our knowledge, this study is the first to describe risk factors for the three main viral pathogens causing gastroenteritis and to estimate the effect of these risk factors in the population. The main risk factor for NV, SLV, and rotavirus gastroenteritis was contact with persons with gastroenteritis, supporting the hypothesis that these viruses are mainly transmitted from person to person (*13*,*15*). The high PARs indicate that most of these infections can indeed be prevented by stopping the spread from symptomatic persons to others.

Food-handling hygiene in the household was also strongly associated with risk for NV gastroenteritis and with a high PAR. This association indicates that in a household setting these viruses do not necessarily transmit directly from one person to another but by means of food. Hygienic food-handling procedures can therefore further prevent the infection spreading from one person to another (*16*).

The impact of food-handling hygiene can be partly explained by food contamination that occurs when a sick household member prepares meals. However, food contaminated at an earlier step in the food chain may also be a source. On the basis of our data, an estimated 12%-16% of NV gastroenteritis and 4% of rotavirus gastroenteritis cases are caused by introduction of contaminated food or water. This figure may be an overestimate, if infection through the shedding of other asymptomatic persons plays a major role, or if the knowledge of respondents about illness in their contacts is limited. Alternatively, the proportion of NV infections attributable to foodborne transmission might be an underestimate since we assumed that, if contact with symptomatic others had taken place, such contact was always the cause of illness. The 40% of NV infections that were foodborne, as presented by Mead (*17*) on the basis of NV outbreak surveillance, is higher than our 12%-16% estimate related to contaminated food’s entering the household and comparable with the 47% related to food hygiene. Mead’s estimate was extrapolated to a community incidence from preliminary NV data from our community study (8,17). Clearly, the precise number of community cases of viral foodborne infection cannot be derived by either approach. However, we strongly support the conclusion that a considerable proportion of NV infections may be prevented by improving food hygiene.

In surveillance systems of outbreaks of NV, person-to-person spread and foodborne spread are reported to be the most common transmission routes (*1*,*8*,*18*). The relative importance of each differs by country and is strongly influenced by the design of the surveillance system (*19*). Outbreaks covered in a surveillance system do not necessarily represent all outbreaks. Our study shows that in sporadic cases, direct and indirect person-to-person transmission remains the most prominent mode of transmission, followed by food contaminated outside the household. Nevertheless, extrapolation of our estimate to the population of the Netherlands (16 million) suggests that, of the 650,000 NV gastroenteritis cases that occur annually, an estimated 80,000 cases are foodborne, which is more than the estimate for Salmonella (50,000 foodborne cases each year).

No specific food products were associated with NV gastroenteritis. This finding is not remarkable because NV can probably survive on almost all food products that are not cooked before consumption, and a very low infectious dose is required, as has been demonstrated for another naked single-stranded RNA virus, poliovirus (*20*). Since NV cannot be grown in cell culture, little is known about its heat inactivation profiles. However, studies for another enteric single-stranded RNA virus with similar structure (hepatitis A virus) suggest that heating for 30 s at 90°C will completely inactivate viruses in any food (*21*). Most published foodborne outbreaks could be traced back to infected food handlers at some point in the production chain, suggesting that this is by far the most common source of foodborne infections (*8*,*22*–*24*). Our results show that, without applying extraordinary hygienic practices but by just following normal hygiene procedures, a substantial portion of sporadic NV infections could be prevented. Because of the high transmission rate in households, persons with a household member with gastroenteritis are at greater risk of being infected. Since several foodborne outbreaks have been reported in which the food handler who had most likely contaminated the food was not symptomatic (yet), making professional food handlers aware of their higher probability of being infected when living with a household member with gastroenteritis might be useful (*24*).

For NV, for persons of >5 years, PAR for food-handling hygiene and the combined PAR for food-handling hygiene and a household gastroenteritis contact were similar. The decrease in OR for a household gastroenteritis contact to almost 1, after hygiene was included in the model, suggests that transmission from one ill household member to another occurs almost entirely through food in persons of >5 years. For children, only part of the transmission from one person to another in the household is through food-handling hygiene. Possibly, for young children, exposure is very common and better food-handling hygiene in the household only prevents a minority of exposure possibilities.

A large study in U.S. households showed that the proportion of rotavirus infections acquired in the household was higher for adults than for children, indicating that children introduce the infection into the household (*25*). In contrast to rotavirus and SLV gastroenteritis, NV gastroenteritis is not limited to the youngest age groups. This finding could explain why food-handling hygiene and having a household gastroenteritis contact had a higher impact on NV gastroenteritis than on SLV gastroenteritis. For rotavirus and SLV, undetected asymptomatic infections (not included as cases in this study) may occur at older age through these routes.

Living in a household with a child attending a daycare center or primary school was univariately associated with NV gastroenteritis. However, when the data were corrected for a household gastroenteritis contact, especially with a child, this association disappeared. This finding suggests that daycare centers and primary schools are the settings in which the primary infection in the household was acquired. The fact that a participant’s daycare attendance had only borderline association with NV gastroenteritis indicates that gastroenteritis acquired at daycare centers was less common in our study than secondary transmission in the household.

We could not confirm the association between rotavirus gastroenteritis and daycare center attendance, as has been reported by others (*26*,*27*). A study with comparable methods in England also did not find this association (*28*).

### Methodologic Issues

The optimized score for food-handling hygiene was fitted in the same NV data for which it was used to estimate the effect of hygiene. This might have resulted in an overestimate for NV because noise is also modeled in the prediction. Although all the factors included in the food-handling hygiene index are related to food handling, we cannot exclude the possibility that the index might be a proxy for hygiene as a whole and not just related to food handling. Finally, we assumed that the relationship between the hygiene score and the risk for NV gastroenteritis was exponential. Although this assumption is not entirely true, the exponential model was a good approximation.

In addition, the uncertainty around the estimates will be wide, and PARs should be interpreted as indications of the magnitude of the effect of a risk factor. Many risk factors were tested for, and a type 1 error might have occurred, identifying risk factors that were in fact not associated to the disease. However, for most of the risk factors, plausible biological mechanisms exist. An exception is the association of a blender with rotavirus, on which, therefore, no conclusions are drawn.

By using a case-control design based on clinical gastroenteritis, differentiating risk factors for infection with the virus from risk factors for developing illness after infection is not possible. Factors that represent long-term exposure might have induced immunity earlier in life, and subsequent infections might not result in clinical disease (*29*,*30*). As a result, long-term risk factors might be more difficult to detect in case-control studies, or, if the proportion of individual persons with immunity is large, even cause a negative association. Especially for rotavirus, for which immunity is proven to exist, this factor might play a role. Whether relevant immunity to NV and SLV is induced is still under debate.
